# The Role of Sugar Transporter Genes during Early Infection by Root-Knot Nematodes

**DOI:** 10.3390/ijms19010302

**Published:** 2018-01-19

**Authors:** Dan Zhao, Yang You, Haiyan Fan, Xiaofeng Zhu, Yuanyuan Wang, Yuxi Duan, Yuanhu Xuan, Lijie Chen

**Affiliations:** 1 College of Plant Protection, Shenyang Agricultural University, Dongling Road 120, Shenyang 110866, China; zhaodan_1201@syau.edu.cn (D.Z.); youyang2015@syau.edu.cn (Y.Y.); fanhaiyan6860@gmail.com (H.F.); syxf2000@syau.edu.cn (X.Z.); duanyx@syau.edu.cn (Y.D.); 2 College of Biotechnology, Shenyang Agricultural University, Dongling Road 120, Shenyang 110866, China; wangyuanyuan2018@gmail.com

**Keywords:** sugar transporter gene, soluble sugar, tomato, *Arabidopsis thaliana*, root-knot nematode

## Abstract

Although pathogens such as nematodes are known to hijack nutrients from host plants, the mechanisms whereby nematodes obtain sugars from plants remain largely unknown. To determine the effects of nematode infection on host plant sugar allocation, soluble sugar (fructose, glucose, sucrose) content was investigated using high-performance liquid chromatography with refractive index detection and was found to increase significantly in tomato (*Solanum lycopersicum*, *Sl*) leaves and roots during early infection by root-knot nematodes (RKNs). To further analyze whether sugar transporters played a role in this process, the expression levels of sucrose transporter (*SUT/SUC*), Sugars Will Eventually be Exported Transporter (*SWEET*), tonoplast monosaccharide transporter (*TMT*), and vacuolar glucose transporter (*VGT*) gene family members were examined by qRT-PCR analysis after RKN infection. The results showed that three *SlSUTs*, 17 *SlSWEETs*, three *SlTMTs*, and *SlVGT1* were upregulated in the leaves, whereas three *SlSUTs*, 17 *SlSWEETs*, two *SlTMTs*, and *SlVGT1* were induced in the roots. To determine the function of the sugar transporters in the RKN infection process, we examined post-infection responses in the *Atsuc2* mutant and *pAtSUC2-GUS* lines. β-glucuronidase expression was strongly induced at the infection sites, and RKN development was significantly arrested in the *Atsuc2* mutant. Taken together, our analyses provide useful information for understanding the sugar transporter responses during early infection by RKNs in tomato.

## 1. Introduction

The root-knot nematodes (RKNs) *Meloidogyne incognita* are sedentary endoparasitic nematodes that mainly infect the roots of host plants [1]. Second-stage juveniles (J2s), the only infective stage, migrate through intercellular spaces to reach the vascular cylinder [2]. Once they reach a suitable site in the host roots, J2s select several single cells and induce specialized nematode feeding sites. These sites become enlarged and contain multinucleated cells termed giant cells (GCs) [3]. During juvenile development, GCs act as nutrient source tissues, providing the necessary nourishment to the nematodes [4]. In the syncytia which were induced by cyst nematodes, such as *Heterodera schachtii*, an increase in metabolic activity has been observed also in relation to soluble sugars [5]. However, little is known about the changes in soluble sugars in GCs. In most plant species, soluble sugars mainly include fructose, glucose, and sucrose [6]. Recently, several members of gene families encoding mono- or disaccharide transporters have been characterized, such as sucrose transporter (*SUT*), Sugars Will Eventually be Exported Transporter (*SWEET*), tonoplast monosaccharide transporter (*TMT*), and vacuolar glucose transporter (*VGT*). These sugar transporter genes play key roles in the regulation of plant development and plant responses to pathogens [7,8,9,10].

Since the identification of *SoSUT1* in spinach (*Spinacia oleracea* L.), many genes in the *SUT* family have been isolated from several higher plants [11,12,13,14,15,16]. Previous studies have shown that *SUTs* play a key role in plant-pathogen interactions. For example, in maize (*Zea mays*), the expression of *ZmSUT1* is significantly altered in response to infection by *Colletotrichum graminicola* [17]. Similarly, in cucumber (*Cucumis melo*), *CmSUT1* is activated upon cucumber mosaic virus (CMV) infection in the leaves [18]. Furthermore, *AtSUC2* and *AtSUC4* are expressed in the syncytia induced by *Heterodera schachtii* in *Arabidopsis thaliana* [19,20].

The plant SWEET family is a recently identified family of sugar transporters, which has seven transmembrane helices in eukaryotes [21,22]. According to phylogenetic studies in several plants, SWEET proteins can be classified into four clades [23,24,25,26,27,28]. Among these, Clades I and II SWEETs typically show a preference for glucose, whereas Clade III SWEETs transport sucrose [21,29]. Clade IV SWEETs are located in the tonoplast, where they are probably involved in the transport of fructose [30,31]. Many studies have shown that *SWEETs* play a vital role in plant–pathogen interactions [21]. In *Arabidopsis*, *AtSWEET2* encodes a vacuolar glucose transporter, which provides an efficient nutrient-based defense mechanism that contributes to resistance against *Pythium irregulare* [32]. In grapes (*Vitis vinifera*), *VvSWEET4*, which acts as a glucose transporter, is highly induced after infection by the necrotroph *Botrytis cinerea* [27]. More recently, in sweet potato (*Ipomoea batatas*), *IbSWEET10* was demonstrated to have considerable potential in improving resistance to *Fusarium oxysporum* [33].

TMTs are localized in the tonoplast and potentially act as H^+^/sugar antiporters, transporting glucose and fructose into the vacuoles [34]. Under drought, salt, and cold stress, *TMT1* and *TMT2* were induced in *Arabidopsis* plants [35]. The *VGT* family members have the same functions as *TMTs*, as evidenced by their expression in similar tissues and their preference for transporting glucose [36]. Although the functions of *TMTs* and *VGTs* have previously been characterized under abiotic stress [35,37], the roles of the sugar transporter gene families in tomato–RKN interactions remain poorly understood.

In this study, the soluble sugar content and the expression of several sugar transporter gene family members were investigated during early infection by RKNs. We demonstrated that the expression of sugar transport genes and the soluble sugar content were altered by RKN infection. We further demonstrated that, whereas the mutation of *AtSUC2* did not inhibit RKN invasion, it did lead to a significant delay in RKN development. Collectively, our results indicate the important role of sugar transport during early infection by RKNs and enhance our understanding of *AtSUC2* function in plant–nematode interactions.

## 2. Results

### 2.1. Accumulation of Soluble Sugar during Early Infection by Root-Knot Nematodes

**Figure 1:**
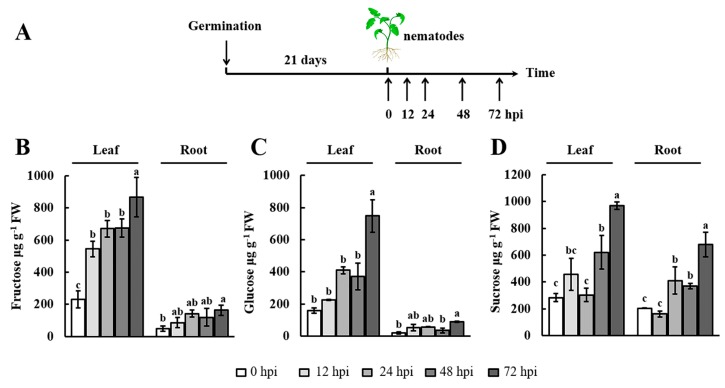
Soluble sugar content in the leaves and roots after nematode infection. (**A**) Scheme of leaf and root sampling for soluble sugar measurements; (**B**–**D**) The soluble sugar content was monitored by high-performance liquid chromatography with refractive index detection (HPLC–RID) in leaves and roots at 0, 12, 24, 48, and 72 h postinoculation (hpi) with *Meloidogyne incognita*. The experiments were repeated three times. Data represent the means ± SE (*n* = 3). Significant differences (*p* < 0.05) between the groups are indicated by different letters. SE, standard error. FW, fresh weight.

Given that RKNs depend entirely on the host root for nourishment, changes in soluble sugar content may reflect the survival demands of RKNs at different points in time [38]. Generally, the accumulation of soluble sugars in the leaves is dependent on photosynthesis and transport activity from the leaves to the roots [39]. Hence, we examined changes in the soluble sugar (fructose, glucose, and sucrose) contents in both the leaves and roots of 21-day-old tomato plants. In this experiment, the samples were collected at 0, 12, 24, 48, and 72 h postinoculation (hpi) with RKNs (Figure 1A). The results showed that the fructose and glucose content increased marginally from 0 to 48 hpi in the leaves and roots, whereas they were increased significantly at the 72 hpi time point. In addition, compared with 0 h, the sucrose content showed a clear increase at 24 hpi in the roots. Moreover, the maximum sucrose content in both leaves and roots was recorded at 72 hpi. Notably, similar trends in fructose, glucose, and sucrose accumulations were observed in the leaves and roots. These results indicated that the soluble sugar content was modulated by early infection with RKNs.

### 2.2. Analysis of SlSUT Expression during Early Infection by Root-Knot Nematodes

**Figure 2:**
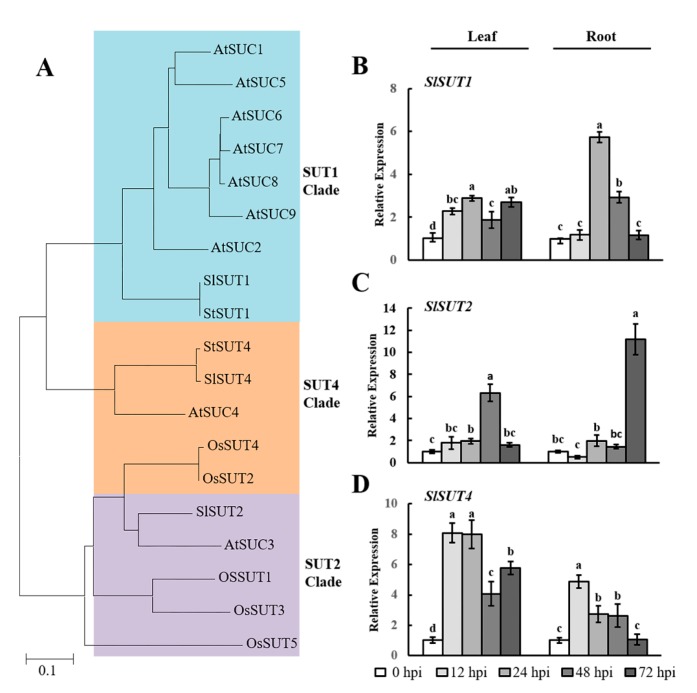
RKN infection-mediated expression of *SlSUTs* in tomato leaves and roots. (**A**) Phylogenetic analysis of SUT proteins from *Arabidopsis thaliana* (*At*), *Oryza sativa* (*Os*), *Solanum tuberosum* (*St*), and *Solanum lycopersicum* (*Sl*). A phylogenetic tree was constructed via the neighbor-joining method with 1000 bootstrap replications. Accession numbers are listed in Supplementary Table S1; (**B**–**D**) qRT-PCR was used to quantify *SlSUT* expression at 0, 12, 24, 48, and 72 h postinoculation (hpi) with *Meloidogyne incognita*. Three biological replicates were analyzed and three technical repeats were performed per sample. Error bars indicate the standard deviation (SD) between biological repeats (*n* = 3). Different letters indicate significant differences (*p* < 0.05).

SUT family proteins function as key sucrose transporters [24]. We initially studied the phylogenetic relationships between SUT sequences in *Arabidopsis thaliana*, *Oryza sativa*, *Solanum tuberosum*, and *Solanum lycopersicum* (*Sl*) (Figure 2A). The SUT sequences in tomato can be classified into three broad classes: SlSUT1, SlSUT2, and SlSUT4. Next, qRT-PCR analysis was used to quantify the expression of *SlSUTs* during early infection by RKNs. The expression of *SlSUT1* was induced by RKN infection and reached a maximum at 24 hpi in the leaves (~2.9-fold) and roots (~5.7-fold) (Figure 2B). As shown for *SlSUT2*, the maximum expression was recorded at 48 hpi (~6.3-fold) and 72 hpi (~11.2-fold) in the leaves and roots, respectively (Figure 2C). *SlSUT4* was strongly expressed at 12 hpi and subsequently maintained a trend of downregulation in both tissues (Figure 2D). These analyses revealed that the expression of *SlSUTs* was highly induced in the leaves and roots at different points of time, suggesting that *SlSUTs* could play an important role during early infection by RKNs.

### 2.3. Analysis of SlSWEET Expression during Early Infection by Root-Knot Nematodes

**Figure 3:**
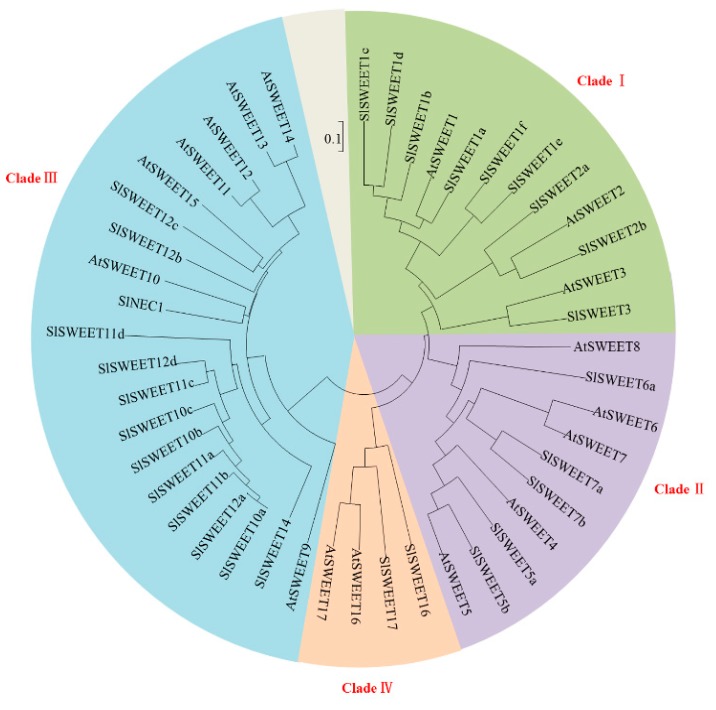
Phylogenetic analysis of SWEET proteins. The conservation of SWEET proteins from *Arabidopsis thaliana* (*At*) and *Solanum lycopersicum* (*Sl*) was assessed by phylogenetic tree analysis. A phylogenetic tree was constructed via the neighbor-joining method with 1000 bootstrap replications. The accession numbers are listed in Supplementary Table S1.

**Figure 4:**
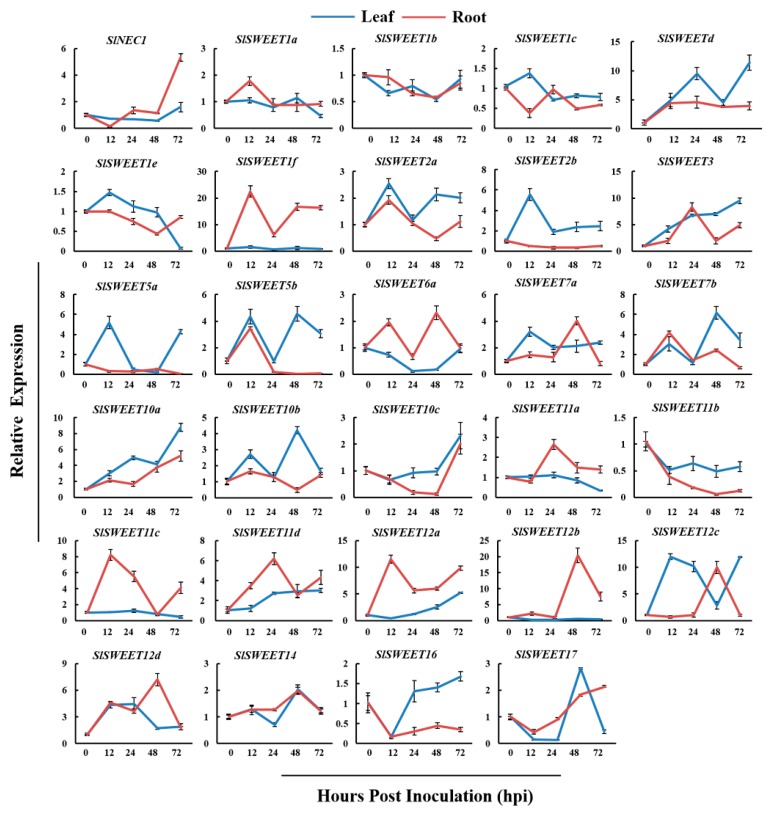
RKNs infection-dependent expression of *SlSWEETs* in tomato leaves and roots. qRT-PCR was used to quantify *SlSWEET* expression at 0, 12, 24, 48, and 72 h postinoculation (hpi) with *Meloidogyne incognita*. Three biological replicates were analyzed and three technical repeats were performed per sample. Error bars indicate the SD between biological repeats (*n* = 3).

Recently, it has been reported that the SWEET family members play a key role in sugar transport [40]. In tomato, 29 SWEETs have been identified and can be classified into four phylogenetic clades [25], which is similar to the pattern previously reported in *Arabidopsis* [21] (Figure 3). To investigate the roles of *SlSWEETs* during early infection by RKNs in tomato, the expression of *SlSWEETs* in the leaves and roots was determined by qRT-PCR. In the leaves, among all *SlSWEET*s examined, eight genes (*SlSWEET1d*, *-2a*, *-2b*, *-3*, *-5a*, *-5b*, *-7a*, *-7b*) in Clades I and II, eight genes (*SlSWEET10a*, *-10b*, *-10c*, *-11d*, *-12a*, *-12c*, *-12d*, *-14*) in Clade III, and *SlSWEET17* in Clade IV were upregulated (>2-fold) at least at one time point, whereas the expression of other genes was downregulated or remained unchanged (Figure 3 and Figure 4). In the roots, eight genes (*SlNEC1*, *SlSWEET1d*, *-1f*, *-3*, *-5b*, *-6a*, *-7a*, *-7b*) in Clades I and II, eight genes (*SlSWEET10a*, *-11a*, *-11c*, *-11d*, *-12a*, *-12b*, *-12c*, *-12d*) in Clade III, and *SlSWEET17* in Clade IV showed enhanced expression (>2-fold), whereas other genes were downregulated or remained unchanged (Figure 3 and Figure 4). Most notably, 10 genes (*SlSWEET3*, *-5a*, *-7a*, *-7b*, *-10a*, *-11d*, *-12a*, *-12c*, *-12d*, *-17*) were significantly upregulated in both leaves and roots. Overall, these data showed that the expression of *SlSWEETs* was dramatically altered, which suggests an important role of these genes during early infection by RKNs.

### 2.4. Analysis of TMT and VGT Expression during Early Infection by Root-Knot Nematodes

**Figure 5:**
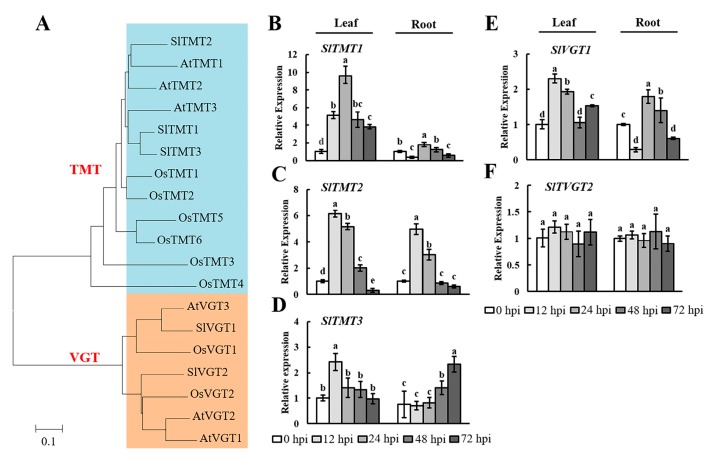
Expression levels of *SlTMTs* and *SlVGTs* in tomato leaves and roots after RKN infection. (**A**) Phylogenetic analysis of TMT and VGT proteins from *Arabidopsis thaliana* (*At*), *Oryza sativa* (*Os*), and *Solanum lycopersicum* (*Sl*). A phylogenetic tree was constructed using the neighbor-joining method with 1000 bootstrap replications (TMT: blue part; VGT: yellow part). The accession numbers are listed in Supplementary Table S1. qRT-PCR was used to quantify *SlTMT*; (**B**–**D**) and *SlVGT*; (**E**, **F**) expression at 0, 12, 24, 48, and 72 h postinoculation (hpi) with *Meloidogyne incognita*. Three biological replicates were analyzed and three technical repeats were performed per sample. Error bars indicate the SD between biological repeats (*n* = 3). Different letters indicate significant differences (*p* < 0.05).

Plants have several small gene families that encode mono- or disaccharide transporters at the plasma membrane or tonoplast [35,41]. Therefore, we selected the *TMT* and *VGT* gene families as targets and examined their expression in tomato leaves and roots during early infection by RKNs. TMT and VGT proteins form small subfamilies with three and two members in tomato, respectively (Figure 5A). The results showed that the expression of these genes was enhanced and inhibited at different levels. In the leaves, *SlTMTs* were upregulated at 12, 24, and 48 hpi (Figure 5B–D). In the roots, *SlTMT1* and *SlTMT2* were upregulated significantly at 12 and 72 hpi, respectively. Additionally, *SlTMT2* was upregulated significantly in both the leaves and the roots at 12 hpi. The analysis of *SlVGTs* showed that *SlVGT1* was significantly expressed in the leaves and roots (Figure 5E). In contrast, there was no significant difference in the expression of *SlVGT2* (Figure 5F). These results indicated that *SlTMTs* and *SlVGT1* could play important roles in the response to early RKN infection in tomato.

### 2.5. The Role of AtSUC2 during Early Infection by Root-Knot Nematodes

**Figure 6:**
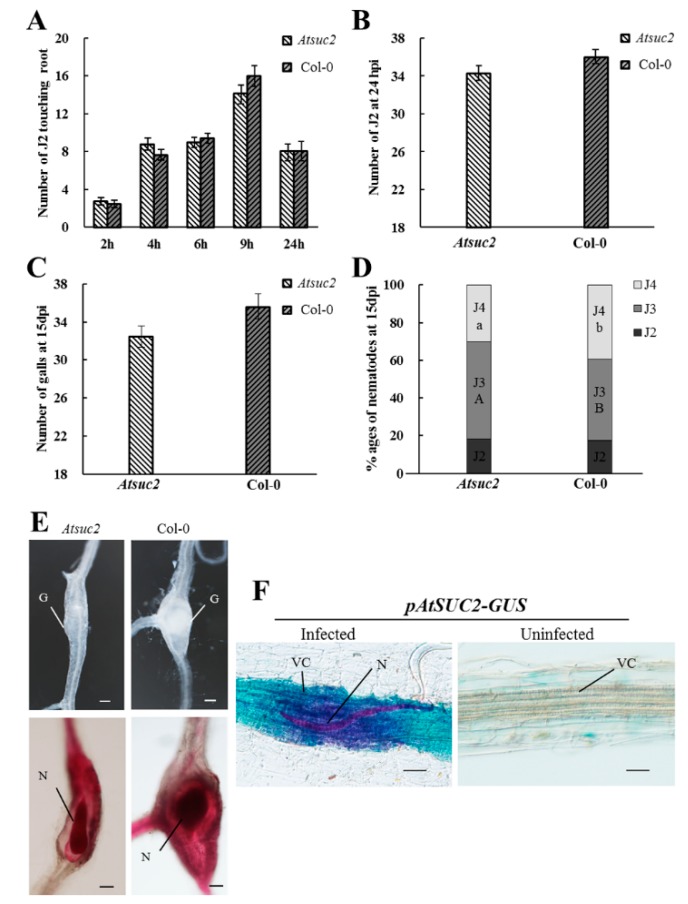
Response of *Arabidopsis thaliana suc2* mutants and wild-type Col-0 plants to *Meloidogyne incognita* infection. (**A**) Second-stage juveniles (J2s) touching the terminal 7 mm of the root of the *Atsuc2* mutant and wild-type Col-0 plants were counted at 2, 4, 6, 9, and 24 h in Pluronic F-127 gel (*n* = 20); (**B**) Number of nematodes inside the roots of the *Atsuc2* mutant and wild-type Col-0 plants at 24 h post-inoculation (hpi) (*n* = 30); (**C**) Number of galls at 15 days postinoculation (dpi) (*n* = 30); (**D**) Percentage of nematodes corresponding to different developmental stages (J3, third-stage juvenile; J4, fourth-stage juvenile) in the *Atsuc2* mutant and wild-type Col-0 at 15 dpi (*n* = 30). Different letters indicate statistically significant differences when compared with the wild-type Col-0. (**A**) or (**B**) indicate significant differences in the J3 stage; a or b indicate significant differences in the J4 stage; (**E**) Galls and acid fuchsin-stained nematodes in the *Atsuc2* mutant and wild-type Col-0 plants. G, gall; N, nematode. Scale bars = 50 µm; (**F**) GUS activity was observed in vascular cylinders at 24 hpi. N, nematode; VC, vascular cylinder. Scale bars = 50 µm. Data represent the means ± SE. The data of plants in each experiment were analyzed using a *t*-test for independent samples (*p* < 0.05). Similar results were obtained when these experiments were performed in duplicate. SE, standard error.

*Arabidopsis* has been established as an important model system for studying the function of plant genes. The *AtSUC2* gene in *Arabidopsis* has high homology with *SlSUT1* in tomato [42,43]. Our results revealed that the expression of *SlSUT1* increased significantly in tomato roots at 24 hpi (Figure 2C). To study the function of *SlSUT1*, we investigated the role of *AtSUC2* in RKN attraction, invasion, and development after inoculation. To evaluate the attraction of RKNs near the roots, we counted the number of J2s touching the root tips of the *Atsuc2* mutant and wild-type Col-0 plants at 2, 4, 6, 9, 12, and 24 h. The number of J2s touching the roots changed over time, with the highest number being observed at 9 h (Figure 6A). Furthermore, the number of J2s around the roots was dramatically lower at 24 h, probably because J2s had already invaded the roots at this time point. There was no significant difference in the number of J2s attracted to the roots of the *Atsuc2* mutant and wild-type Col-0 plants. Fudali et al. [44] observed that at 24 hpi, a large number of RKNs had invaded the roots. Therefore, we selected 24 h as a suitable time point to perform the invasion assay. Our results showed that there was no significant difference between the *Atsuc2* mutant and wild-type Col-0 plants in terms of the number of J2s found in the roots (Figure 6B). To further investigate the role of *AtSUC2* during gall formation and RKN development, the number of galls and different stages of RKNs were counted at 15 days postinoculation (dpi). The results showed that there was no difference in the number of galls in the *Atsuc2* mutant when compared with the wild type Col-0 (Figure 6C). The analysis of the *Atsuc2* mutant showed that the mutation of *AtSUC2* led to a lower proportion of fourth-stage juveniles (J4s) than in the wild-type Col-0, and most of the nematodes remained in the third-stage juveniles (J3s) (Figure 6D,E). These results indicated that the mutation of *Atsuc2* did not inhibit RKN invasion, but did result in a significant delay in RKN development.

To analyze the regulation of *AtSUC2* in RKN infection, β-glucuronidase (GUS) assays were performed at 24 hpi. An intensive GUS signal was observed in the infected areas of the roots, particularly in the vascular cylinder in *Arabidopsis* (Figure 6F). The signal remained at a plateau level and was restricted to an area close to the RKN head. The GUS signal was not found in uninfected roots. These results indicated that RKN infection activated the expression of *AtSUC2* in *Arabidopsis* roots.

## 3. Discussion

Pathogens infect plants in order to hijack host nutrients, particularly sugars [21]. Accordingly, the expression of sugar transporter genes is altered in plants after infection by pathogens. Recently, phylogenetic and functional analyses of sugar transporter gene families (*SUT*, *SWEET*, *TMT*, and *VGT*) have been conducted for many plant species [36,45,46]. To date, however, there have been no reports on the regulation of soluble sugars and the expression of sugar transporter genes in tomato–RKN interactions. In the present study, we observed a dramatic increase in the soluble sugar contents of tomato leaves and roots during early infection by RKNs, with the expression of sugar transporter genes being induced at the same time points. Furthermore, the functional analysis of *AtSUC2* demonstrated a strong effect on RKN development. This study revealed the regulation of soluble sugar content and sugar transporter genes in tomato, and enhances our current understanding of *AtSUC2* function in *Arabidopsis* during infection by RKNs.

A previous study revealed that infection by *M. incognita* alters host plant metabolism, particularly photosynthesis [47]. Therefore, gaining an understanding of the regulation of sugar metabolism and transport will provide a theoretical basis for the prevention of nematode disease. During nematode infection, sugar contents undergo marked changes in *Arabidopsis* [5], and soluble sugars have been described as playing significant roles during cyst nematode infection [48]. Consistent with the findings of previous research, in our study, we found that the soluble sugar content increased in tomato leaves and roots after RKN infection (Figure 1). At 12 hpi, J2s induced the first GC and started feeding [49]. In this study, we observed a slight increase in the soluble sugar content in the leaves and roots at 12 hpi (Figure 1). During the formation of GCs, the amounts of available soluble sugars must be sufficient to support nematode survival. Our results showed that the soluble sugar content in the leaves and roots dramatically increased to a maximum at 72 hpi (Figure 1). These results underline the importance of soluble sugars in meeting RKN energy requirements during early infection in tomato.

SUT family members are well established as H^+^/sucrose symporters [50]. In this study, the sucrose content increased during early infection by RKNs (Figure 1D). This result implied that sucrose could be an important energy source for RKNs. We also observed that the expression of *SlSUT*s was increased during early infection by RKNs (Figure 1C–E). Our findings were consistent with previous studies that reported the importance of *AtSUCs* in nematode infection in *Arabidopsis* [4,19,51]. To further explore the function of *SUT1*, we analyzed the function of *AtSUC2* in *Arabidopsis*, which has high homology to *SlSUT1* in tomato. The successful infection of plant roots by RKNs necessitates completion of the following phases: effective attraction toward the root tips, establishment of a feeding site, and absorption of nutrients for development [51]. We evaluated these three steps to characterize the role of *AtSUC2*. On the basis of our results, we concluded that the mutation of *AtSUC2* did not inhibit RKN invasion (Figure 6A–C), but did result in a significant delay in RKN development (Figure 6D,E). The mutation of *Atsuc2* in *Arabidopsis* may reduce sucrose translocation in the roots, and, under such circumstances, RKNs would be unable to absorb adequate nutrients to support development. The histochemical analysis using *pAtSUC2-GUS* transgenic plants revealed that the *AtSUC2* gene was induced in the early infection process (24 hpi) at the site of RKN infection (Figure 6F). At 24 hpi, RKNs had invaded throughout the roots and had begun establishing feeding sites [52]. It has been suggested that *AtSUC2*, as a phloem-specific sucrose transporter, may be crucial for sucrose import into GCs, particularly at 24 hpi. A previous study showed that GUS expression was not observed in the feeding cells induced by RKNs in *pAtSUC2*-*GUS* plants at 7 dpi [19]. Hence, there may be other sucrose transporter genes that contribute to sucrose availability for RKNs at 7 dpi, which is a stage during RKN development when J2 and J3 individuals are dominant. However, it remains to be determined which genes are involved in this process and how they control sucrose accumulation. Accordingly, further studies are needed to elucidate the role of sucrose transporter genes covering the entire life cycle of RKNs.

Plant SWEET proteins are grouped into four clades based on amino acid homologies [25]. Many studies have shown that *SWEETs* play an important role in plant–pathogen interactions. Clade I and II members function as plasma membrane glucose transporters. In rice (*Oryza sativa*), expression analyses have shown a clear induction of *OsSWEET3b* by arbuscular mycorrhizal [28]. In *Arabidopsis*, *AtSWEET5* and *-7* have been found to be highly induced by *Pseudomonas syringae* pv. *tomato* strain DC3000 [21]. Similarly, in the present study, the expression of *SlSWEET3*, *-5b*, *-7a*, and *-7b* was highly induced after RKN infection (Figure 4), implying that these genes may play roles during RKN infection or development. Clade III SWEETs have been identified as key factors in sucrose efflux from phloem parenchyma cells [29]. In *Arabidopsis*, *AtSWEET10* expression is induced by *Pythium* infection in roots [32]. Moreover, *AtSWEET12* expression is upregulated by infection with *Golovinomyces cichoracearum* [21]. In rice, *OsSWEET11* is a target of *Xanthomonas oryzae* pv. *oryzae*, which probably provides sugars to the site of infection [53]. Consistent with the finding of previous studies, we showed that the expression of *SlSWEET10a*, *-11d*, *-12a*, *-12c*, and *-12d* followed a similar trend as the changes in sucrose content (Figure 1C and Figure 4). This evidence strongly supports the importance of Clade III SWEETs in the change of sucrose as a source of energy during RKN infection. SWEET17, a member of Clade IV, plays a key role in exporting fructose in *Arabidopsis* [30,31]. Concomitant with an increase in fructose content, *SlSWEET17* was highly expressed in both the leaves and the roots (Figure 1B and Figure 4). The results of the gene expression analysis showed that various *SlSWEETs* could be induced by RKNs and played a significant role during early infection by RKNs. In future experiments, it will be of interest to determine the function of these genes in plant–RKN interactions.

In *Arabidopsis*, TMTs are a type of vacuolar carrier proteins with transport capacity for both glucose and fructose [35]. *TMT1* is expressed in *Arabidopsis* roots infected by *M. incognita*. [54]. In the present study, *SlTMTs* were strongly expressed during early infection by RKNs, a period that coincides with the phase of glucose and fructose accumulation (Figure 1B,C and Figure 5B–D). Members of the *VGT* gene family, which is similar to the *TMT* gene family, play roles in the transport of glucose [37]. In the present study, *SlVGT1* was induced during early infection by RKNs, whereas *SlVGT2* did not appear to be involved in the change in glucose content in the leaves and roots (Figure 1C and Figure 5E,F). On the basis of these observations, we speculated that *SlTMTs* and *SlVGT1* played an important regulation in the accumulation of glucose and fructose during early infection by RKNs. Further studies are required to elucidate the mechanisms underlying the activities of *SlTMTs* and *SlVGT1* in tomato–RKN interactions.

In conclusion, the soluble sugar content was increased in tomato leaves and roots during early infection by RKNs. The sugar content increase in the leaves may be a result of the activation of photosynthesis or the hydrolysis of starch promoted by environmental changes, e.g., RKN infection, whereas an increase in sugar in the roots may be a consequence of sugar translocation from source to sink tissue via transporters. A higher induction of sugar transporter genes might be linked to this process, which will be interesting to examine in further studies. Previous studies have reported that SUT and Clade III SWEET proteins transport sucrose. The excessive accumulation of sucrose may result from the activities of three *SlSUTs* and 11 *SlSWEETs* in Clade III (*SlSWEET10a*, *-10b*, *-10c*, *-11a*, *-11c*, *-11d*, *-12a*, *-12b*, *-12c*, *-12d*, *-14*). In addition, TMT and Clade IV SWEET proteins transport fructose. Three *SlTMTs* and *SlSWEET17* appear to be involved in changes in fructose content. Whereas VGT and Clade I/II SWEET proteins transport glucose, six *SlSWEETs* in Clade I (*SlNEC1*, *SlSWEET1d*, *-1f*, *-2a*, *-2b*, *-3*), five *SlSWEETs* in Clade II (*SlSWEET5a*, *-5b*, *-6a*, *-7a*, *-7b*), and *SlVGT1* may be required for the accumulation of glucose. Furthermore, *AtSUC2* was shown to play an important role in the development of RKNs, providing compelling evidence of the importance of sugar transporters in the success of RKN infection of host plants. The results of our study provide baseline information for further studies aimed at understanding the function of sugar transport genes in plant–RKN interactions.

## 4. Materials and Methods

### 4.1. Nematode Culture and Inoculum Preparation

*M. incognita* was propagated on greenhouse grown tomato, and nematode eggs were collected from 3-month-old heavily galled tomato roots. The roots were washed free of soil with water, cut into 1 cm pieces, and placed in a bottle containing 5.0% (*v*/*v*) sodium hypochlorite (NaOCl). The bottle was then shaken vigorously for 5 min. This suspension was thoroughly rinsed with tap water through a set of sieves with mesh sizes of 250, 150, 75, and 25 µm to remove the NaOCl. The eggs retained on the 25 µm-mesh sieve were collected in tap water. The eggs were subsequently collected using the sucrose flotation technique described by Hussey and Baker [55]. RKNs were extracted using the Baermann funnel method [56] and collected every 24 h.

### 4.2. Plant Materials and Nematode Inoculations

Despite its high susceptibility to nematode attack, L-402 is one of the most popular tomato (*Solanum lycopersicum*) varieties in China. Seeds of L-402 were surface-sterilized in 1% sodium hypochlorite and sown on substrate soil under greenhouse conditions (22–26 °C; 16/8 h, light/dark regime; 70% relative humidity). Twenty-one-day-old plants were transferred into 12 cm plastic pots containing 600 g of soil/sand culture (1:1, *v*/*v*). During the course of the experiment, the plants were watered three times a week and fertilized once a week with half-strength Hoagland nutrient solution. Nematode infection was performed on 21-day-old plants. The plants were inoculated near the root with a mixed population of *M. incognita* at a rate of 1000 per plant. The leaves and roots were harvested at 0, 12, 24, 48, and 72 h after inoculation and frozen in liquid nitrogen for analysis.

To assess the role of *SUC2* during RKN infection and development, *Arabidopsis* wild-type Col-0, the *Atsuc2* mutant [57], and *pAtSUC2-GUS* transgenic plants were used. To generate the *pAtSUC2-GUS* transcriptional construct, 2.5 kb of the *AtSUC2* promoter was amplified by PCR using the primer pair *pAtSUC2* GW-F (5′-GGG GAC AAG TTT GTA CAA AAA AGC AGG CTT CGG TAC CGA TTC ATG TCA CTC CTA GCT AG-3′) and *pAtSUC2* GW-R (5′-GGG GAC CAC TTT GTA CAA GAA AGC TGG GTG ACT AGT TTT GAC AAA CCA AGA AAG TAA G-3′). The PCR product was cloned into pDONR221 using BP clonase (Invitrogen, Carlsbad, CA, USA). After sequencing, the *AtSUC2* promoter fragment was inserted into the pMDC163 GW vector via the LR reaction (Invitrogen) [21,29].

For the attraction assay, the seeds of *Arabidopsis* (wild-type Col-0 and *Atsuc2* mutant) were surface-sterilized in 75% (*v*/*v*) ethanol for 15 min, extensively rinsed with sterilized water, and then placed on plates filled with half-strength Murashige and Skoog medium (1/2 MS) containing 0.8% agar (pH 5.7). The seeded plates were incubated for 2 days at 4 °C and then moved to a culture room under a 16/8 h light/dark regime at 24 °C. After 7 days of growth, *Arabidopsis* plants were removed from the agar plates and placed in PF-127 medium (23% *w*/*v*; Sigma-Aldrich, Saint Louis, MS, USA) containing J2s, as described by Hu et al. [58]. Briefly, the nematode concentration in the PF-127 medium was adjusted to 800 J2s mL^−l^, and then 0.25 mL of this solution was added to each well of a 24-well tissue-culture plate, followed by the placement of one *Arabidopsis* plant into each well. The number of nematodes touching the terminal 7 mm of the root tip was counted at 2, 4, 6, 9, and 24 h.

For RKN penetration and developmental stage assays, *Arabidopsis* plants (wild-type Col-0 and *Atsuc2* mutant) were grown in sand culture in a growth chamber at 22 °C. Plants were maintained under a diurnal cycle of 16 h light–8 h dark throughout the growth period, with light provided by white fluorescent tubes. Two-week-old *Arabidopsis* plants were inoculated with 200 freshly hatched J2s, under the same conditions described previously. At 24 h postinoculation (hpi), the number of nematodes in the roots was estimated using a modified acid fuchsin staining procedure for the RKN penetration assay [59]. At 15 days postinoculation (dpi), the plants were screened, and the gall number was counted for the developmental stage assay. Parasitic J2s subsequently developed into two other juvenile stages, J3 and J4. The number of nematodes in the roots and the different developmental stages were estimated in two independent biological experiments, as previously described.

For GUS assays, *Arabidopsis pAtSUC2-GUS* plants were grown under the same conditions described for the RKN penetration experiment. Infected and uninfected transgenic root tissues were removed from the soil at 24 hpi and infiltrated overnight with GUS-staining buffer containing X-Gluc (Real-Times Biotechnology, Beijing, China), at 37 °C in the dark. The following day, the tissues were washed with 70% ethanol. GUS expression in the roots was monitored microscopically. The photographs were taken using an OLYMPUS BX 53 microscope equipped with an Olympus DP 80 digital camera (Tokyo, Japan).

### 4.3. Soluble Sugar Analysis

The soil was washed off the roots of infected and uninfected plants. Ethanol extracts of leaf and root samples were prepared using the method described by Bethke and Busse [60] and analyzed using a high-performance liquid chromatography system (HPLC; Agilent 1100, Palo Alto, CA, USA) equipped with a refractive index detector (RID) Shodex RI-201H (Showa Denko, Tokyo, Japan) on an amine column (Welch ultimate XB-NH_2_, Shanghai, China), using a Waters chromatography system. HPLC separations were performed using a 75% acetonitrile: 25% alcohol/water mobile phase heated to 30 °C, flowing through the column at a rate of 1.0 mL·min^−l^.

### 4.4. RNA Isolation and Quantitative Reverse Transcription-PCR (qRT-PCR)

Total RNA was extracted using the TRIZOL^®^ reagent (CoWin Biosciences, Beijing, China) according to the instructions of the manufacturer. Prior to reverse transcription, the quality of the RNA samples was determined using a NanoDrop 2000 UV-Vis Spectrophotometer (Thermo Scientific, Waltham, MA, USA). cDNA was used for reverse transcription with an oligo (dT) primer, using the HiScript II Q Select RT SuperMix (Vazyme, Nanjing, China). qRT-PCR was then performed using a BIO-RAD CFX96 real-time PCR system (Bio-rad, Berkeley, CA, USA). The reactions were performed in a total volume of 25 µL using SYBR^®^ Premix Ex Taq™ II (Takara, Tokyo, Japan). All reactions were performed under the following conditions: an initial denaturation step (30 s at 95 °C), followed by 40 cycles of denaturation (5 s at 95 °C), annealing (30 s at 60 °C), and extension (1 min at 72 °C). The cycle threshold (*C*t) values were calculated using CFX Manager 3.0, and Microsoft Office Excel was used to calculate transcript abundance from *C*t values normalized to the actin gene signal. The gene relative expression levels were calculated according to the 2^−ΔΔ*C*t^ method [61]. The primers used for qTR-PCR assays are listed in Supplementary Table S2.

### 4.5. Statistical Analysis

All statistical analyses were performed using the analysis of variance (ANOVA) with the SPSS statistical software package (Version 17.0; SPSS, Inc., Chicago, IL, USA) and Microsoft Office Excel 2010. The differences between the two groups were analyzed using the Student’s *t*-test, whereas the differences among multiple treatments were analyzed using Duncan’s multiple range test. All differences were tested for significance at the *p* < 0.05 level.

## Supplementary Materials

Supplementary materials can be found at www.mdpi.com/1422-0067/19/1/302/s1.

## Abbreviations

*SUT/SUC*	Sucrose transporter
*SWEET*	Sugars will eventually be exported transporter
*TMT*	Tonoplast monosaccharide transporter
*VGT*	Vacuolar glucose transporter
RKN	Root-knot nematode
GC	Giant cell
J2	Second-stage juvenile
J3	Third-stage juvenile
J4	Fourth-stage juvenile
hpi	Hours post-inoculation
dpi	Days post-inoculation
NaOCl	Sodium hypochlorite
MS	Murashige and Skoog medium
GUS	β-Glucuronidase
HPLC	High-performance liquid chromatography
RID	Refractive index detector
qRT-PCR	Quantitative reverse transcription-PCR
