# Supplementary material for: The Role of Sugar Transporter Genes during Early Infection by Root-Knot Nematodes
Source: Int J Mol Sci. 2018 Jan 19;19(1):302. doi: 10.3390/ijms19010302 (PMC5796247; doi:10.3390/ijms19010302)
Supplement: Supplementary file 1 [file ijms-19-00302-s001.zip › Supplemental Table S2.docx]

**Supplemental Table S2 PCR primers used for qRT-PCR assays**

|  | Gene name | Forward primer (5’-3’) | Reverse primer (5’-3’) |
| --- | --- | --- | --- |
| SlSUTs | SlSUT1 | GCTTCCATAGCTGCTGGTGT | GGGTCCACAAAGCCAAATAA |
|  | SlSUT2 | AGCTTGTTGTGAGCCATGTG | GACAGCGGGACTTCATTAGC |
|  | SlSUT4 | CACCATCAACTGTGCCAATC | GGAACCACGACTGGGATCTA |
| SlSWEETs [25] | SlNEC1 | CATTGCGATGCCAAACATTC | TATCTCCACCACCACCGCTT |
|  | SlSWEET1a | TTTGCTGCTGTAGCCCTTGTT | GAACTCCACGCTCTTCGTCTTG |
|  | SlSWEET1b | TGCCTTTCTTCTTGTCCTTG | TGGGTGTTAGATGGCTTCTCT |
|  | SlSWEET1c | TTCCATAGGTGGTGCCTT | CTTTCCCAAGCAAGCCAT |
|  | SlSWEET1d | ATTGCTTGCTCTCAGTTTGG | ACAACGAGGTATCCGCAGA |
|  | SlSWEET1e | GGATTGTTGGAAATGCCGC | GCAGAAAGCAAGCAGTTGAG |
|  | SlSWEET1f | CATTTGCCGCTATCCTATCTTG | TTCCTAAAGTCAACCCAACACC |
|  | SlSWEET2a | TATGCCTCTGGTATGGGACACC | TATCCAACGAAAGTTTGCCGAC |
|  | SlSWEET2b | ATCTGGAACTCCGACGCAT | AGGCACTCATTAGGAAGGTGG |
|  | SlSWEET3 | TCCAAATCTGGTGGGAAC | TCAGTGAGAACGAGCATCG |
|  | SlSWEET5a | AATGGAGGTATTTGGTTCGCCT | TTTCTTCATCGTCCCAGTTGG |
|  | SlSWEET5b | TTGCTGTTTGGATGCGTG | CAATGATTCCTACAACCGTCC |
|  | SlSWEET6a | TCGTTCATCCACACAGCATTC | ATTCCAAAGGCGGTAGCG |
|  | SlSWEET7a | TGATGCCTACATTCTCGCACC | TCCTTTAGCCTCTCTTGCTGCC |
|  | SlSWEET7b | TGCCCTTCTTCCTTTCCCT | CATCCTACCAAACCCACTCC |
|  | SlSWEET10a | TGGTTGGTGGATTTGGTGC | TTGTCTCACGATGCCTAAGGG |
|  | SlSWEET10b | TCCTTTCTCCATTGCCAACG | GCACCAAATCCACCCAC |
|  | SlSWEET10c | GATGGCTATTGCTGGTCATTGG | ACCCTGGCTTTCTTTGGTGC |
|  | SlSWEET11a | CTACGCACCAAAGAAAGCCAG | GTCTCACAATGCCTAAGGGAGC |
|  | SlSWEET11b | CCAACACAACACTTCTCATCAC | GTCCACGAACAACTCCTTTG |
|  | SlSWEET11c | GCACCAAAGAAAGCCAGGGT | CACAAGGCAAATCCATCCAAC |
|  | SlSWEET11d | TCTCGTTCATTGTGTTCCTTGC | TGGACCTTGGCTTTCTTTGG |
|  | SlSWEET12a | ACCAAACAAAGCCAGGGTCC | TCTCACAATGCCTAAGGGTGC |
|  | SlSWEET12b | TGGTCTACACCTTTGGCATT | CCCTTCCGATGATTTCTTCT |
|  | SlSWEET12c | GCATCGTGTTTCAAGTGGTTCG | TCTATCGCTGGCTTTGCGTT |
|  | SlSWEET12d | AAGCCAGGGTCCAAACTGT | GGAATCGCAATGTTGAAGTC |
|  | SlSWEET14 | GCCAAAGGCAATCATAGAGG | AGGCACACACAATCAGACCT |
|  | SlSWEET16 | CTTATGTTCGCTGCTCC | ACTACCATTGACTGTGACCACT |
|  | SlSWEET17 | TGGTGGCGTTTGGACATTGT | GGCAGCAAGTGTTCCGTTTG |
| SlTMTs | SlTMT1 | CAGCGTTCGTGGAATCTGTA | CAGCATAGATGGCGAAGACA |
|  | SlTMT2 | GCAGGGTTGGGATAATGCTA | GACAAGTGTGGCTCCAATGA |
|  | SlTMT3 | CGGCGTCCAATGCTTATTAT | AAATCCATCCAACAGCCTTG |
| SlVGTs | SlVGT1 | TTGGCTCCATACTCGCCTAC | TGGAGCACAGGCAGTTAGTG |
|  | SlVGT2 | AATTGGAATGTGGTGGCTTC | CTAAGCTGGCACAAGCAACA |
| Actin |  | TGTCCCTATTTACGAGGGTTATGC | AGTTAAATCACGACCAGCAAGAT |
